# Medical Marijuana Initiation and Simulated Driving Performance Among Mid-to-Late-Life Adults With Chronic Pain: Prospective Observational Feasibility Cohort Study With Matched Controls

**DOI:** 10.2196/79735

**Published:** 2026-05-08

**Authors:** Nicole Ennis, Yang Hou, Katie Kloss, Jason Rogers, Sherrilene Classen

**Affiliations:** 1 Department of Behavioral Sciences and Social Medicine, College of Medicine, Florida State University, 2010 Levy Ave, Bldg B Suite 266, Tallahassee, FL, 32310, United States, +1 850-644-7955; 2 Department of Occupational Therapy, College of Public Health and Health Professions, University of Florida, Gainesville, FL, United States

**Keywords:** medical marijuana, driving simulator, chronic pain, mid-to-late life, psychomotor function

## Abstract

**Background:**

Marijuana initiation among adults aged 50 years and older has increased substantially. Although acute tetrahydrocannabinol exposure can impair psychomotor function, less is known about how real-world medical marijuana initiation relates to functional tasks such as driving in mid-to-late life.

**Objective:**

The objective of our study was to evaluate the feasibility of recruiting and retaining adults aged 50 years and older, who are newly registered for medical marijuana, and matched non–marijuana-using controls, into a longitudinal high-fidelity driving simulator protocol, and to explore preliminary associations between medical marijuana initiation and simulated driving performance.

**Methods:**

This prospective, nonrandomized feasibility cohort study enrolled adults aged 50 years and older who are newly registered in the Florida Medical Marijuana Use Registry, along with age-, race-, and sex-matched controls. Assessments occurred at baseline (T1; preinitiation) and at 1 month (T2). Primary feasibility outcomes included recruitment, retention, simulator completion and tolerance, and exposure verification. Exploratory outcomes included reaction time and divided attention (DA) performance, which are measured using an immersive, high-fidelity driving simulator.

**Results:**

Recruitment and exposure verification procedures were feasible, but simulator sickness contributed to substantial missing data. Exploratory analyses suggested group differences in select DA outcomes at T2. At T2, reaction time to DA situation 3 (DA3) was significantly shorter in the medical marijuana group (n=14, mean 2.57, SD 1.63) than in the control group (n=7, mean 5.79, SD 4.32; *t*_19_=−2.50, *P*=.02, *g*=−1.11, 95% CI −2.04 to −0.16). These findings should be interpreted cautiously, given the small sample size, missing data, and multiple comparisons.

**Conclusions:**

A prospective protocol examining medical marijuana initiation and simulated driving among mid-to-late-life adults is feasible, but future studies should incorporate design and analytic refinements to address simulator sickness and missing data and to better characterize exposure timing and patterns.

## Introduction

### Known Effects of Acute Use

Evidence shows that acute marijuana use impairs psychomotor functions in real-world tasks such as driving [1]. Current research indicates that, in general, marijuana users experience cognitive impairment in areas such as psychomotor speed, attention, and executive functioning [2-6]. Specifically, research has explored the effect of acute marijuana use on psychomotor skills, which are cognitive motor skills imperative for everyday activities such as safe driving. Researchers have assessed psychomotor function on measures of critical tracking, attention, and reaction time, and the findings have indicated that marijuana usage creates deficits in psychomotor function for up to 3.5 hours after use [7,8]. Impairments to psychomotor abilities have practical implications, as these implications impair an individual’s ability to drive [7,9]. While the acute effects of tetrahydrocannabinol use are known, the effect of medical marijuana use on psychomotor function under real-world conditions remains unknown.

### Older Adults and Psychomotor Function

Research on psychomotor functioning in older adults generally uses cognitive assessment tools, such as the Trail Making Test, which have limited real-world application [10-15]. The test is performed under artificial, time-pressured conditions that do not resemble the self-paced, contextually rich nature of driving performance. Studies of driving performance using high-fidelity driving simulators do mimic real-world conditions and are valid predictors of on-road driving [16-18]. Driving simulators allow for the systematic representation of events and the manipulation of variables (eg, road type, traffic, intersections), which offers experimental control that is dangerous on the road [16,18]. Driving simulators also offer optimal stimulus presentation through realistic and immersive scenarios, which allows for analysis of both healthy and impaired drivers under similar conditions [17]. Using a high-fidelity driving simulator, we can examine response time, attention, and executive functions by simulating valid real-world driving tasks [18]. Medical marijuana use and how it influences driving performance needs to be examined, and the high-fidelity driving simulator offers a safe, reliable, and valid option to do so [19], anticipating future on-road studies to examine these factors under real-world conditions.

### The Current Study

The current study had 2 aims. Aim 1 was to evaluate the feasibility of recruiting and testing adults aged 50 years and older who are newly registered for medical marijuana, along with matched non–marijuana-using controls, in a longitudinal high-fidelity driving simulator protocol [19]. Aim 2 was to explore preliminary associations between medical marijuana initiation and simulated driving performance. Specifically, we examined whether medical marijuana use was associated with changes in divided attention (DA) and reaction time during a driving task among adults aged 50 years and older by assessing participants before and after medical marijuana initiation and comparing them with age-, race-, and sex-matched controls.

## Methods

### Ethical Considerations

The study was approved by the Florida State University Institutional Review Board (IRB; STUDY00001659) and the University of Florida’s IRB (CED000000442). All participants signed an informed consent form prior to enrollment in the study. Study data were coded using participant ID numbers; identifiable data were stored separately from study data on secure, password-protected institutional systems accessible only to authorized study staff. Participants received a US $50 grocery store gift card at each study timepoint. Due to their sensitive nature, data are only available internally to intervention Research Advancing Care Excellence lab staff and related institutional stakeholders (IRB).

### Study Design

This study was a prospective, nonrandomized feasibility cohort study with repeated measures. Adults newly registered for medical marijuana were assessed prior to initiation (T1) and approximately 1 month after initiation (T2). Outcomes were compared with those of age-, race-, and sex-matched non–marijuana-using controls assessed at the same intervals.

Accordingly, the manuscript follows the STROBE (Strengthening the Reporting of Observational Studies in Epidemiology) reporting guidelines for cohort studies. A completed STROBE checklist is provided in Checklist 1.

### Participants

#### Inclusion Criteria

Participants in this study were adults 50 years and older who met the following inclusion criteria: reported chronic nonmalignant pain, had no prior history of medical marijuana use (control group only) or were newly registered in the Medical Marijuana Use Registry [20] in the State of Florida to obtain medical marijuana (medical marijuana group only), engaged in driving regularly (as defined by the individual), could communicate in English, were planning to remain in the area for the duration of their enrollment in the study, and were willing and able to complete all study procedures as per the informed consent form.

#### Recruitment

The medical marijuana group was recruited from the Medical Marijuana Treatment Clinics (MMTC) of Florida’s Gainesville office in Gainesville, FL, USA. The MMTC staff informed patients who completed the process of certification for medical marijuana and met the inclusion criteria about the study either during or prior to their initial clinic visit. The baseline took place before the patients obtained their medical marijuana license, a process that took 10 to 14 business days before making their first purchase at a licensed dispensary. However, on February 1, 2022, the Office of Medical Marijuana Use, which houses the Medical Marijuana Use Registry, began granting immediate card approval to Florida residents, forcing the study team to adapt procedures to mitigate the use prior to the baseline. Specifically, as a result of this regulatory change, we adapted recruitment procedures to explain to participants that the baseline appointment needed to occur before they purchased any products from a dispensary. Furthermore, we adjusted our assessment procedures to accommodate participants within 24 to 72 hours.

The control group was matched for age, race, and sex. Participants in the control group were recruited with support from HealthStreet at the University of Florida, the UF Health Integrated Data Repository within the Clinical and Translational Science Institute at the University of Florida, and recruitment flyers were placed in the community at locations such as coffee shops and grocery stores. All participants received a US $50 Publix (regional grocery store) gift card at each of the 3 timepoints in the study.

### Procedure

#### Study Overview

Eligible participants who provided informed consent were enrolled. Each in-person study appointment lasted for approximately 90 to 120 minutes. All study assessments were conducted in the Smart House at Oak Hammock, a retirement community at the University of Florida (Gainesville, FL). The baseline (T1) assessment for the medical marijuana group participants took place prior to the initiation of medical marijuana, and the second assessment (T2) took place 1 month after medical marijuana initiation. Medical marijuana group participants were contacted through telephone for a Post-Medical Marijuana Initiation Survey to confirm that they had used marijuana, as expected.

The study coordinator administered the Mini-Mental State Examination Second Edition: Brief Version (MMSE-2: BV) to assess the registration, orientation to time, orientation to place, and recall. Next, an audio-recorded marijuana consumption questionnaire was administered to learn about the participants’ previous experiences with marijuana, including history of use, pattern of use, and current use, which enabled us to classify the participants as light, moderate, heavy, or no use.

#### Timeline

This feasibility study was conducted in Gainesville, FL, from September 2020 to August 2023, with active data collection beginning in April 2021 and concluding in August 2023. The parent protocol included 3 planned timepoints: T1, baseline preinitiation; T2, approximately 1 month postinitiation; and T3, approximately 3 months postinitiation. The present feasibility manuscript reports T1 and T2 assessments only.

#### COVID-19 Procedures

The study protocol was modified to incorporate COVID-19 guidelines and recommendations. However, by June 2021, we were able to resume the recruitment of older adults without restrictions, including adults aged 65 years and older.

#### Measures

Potential control and confounding variables were selected to characterize cognitive status, mental health, driving exposure, crash risk, and substance exposure verification. Cognitive screening was assessed using MMSE-2: BV [21]; depressive symptoms were assessed using the Center for Epidemiologic Studies Depression Scale [22]; pain was assessed using the Brief Pain Inventory: Short Form [23,24]; driving exposure and patterns were assessed using the Driving Habits Questionnaire [25]; visual processing or crash risk was assessed using the Useful Field of View [26]; substance use risk or history was assessed using the National Institute on Drug Abuse–modified Alcohol, Smoking, and Substance Involvement Screening Test [27]; recent substance exposure was assessed using urine toxicology [28]; and simulator tolerance was assessed using the Brooks Simulator Sickness Questionnaire [29]. Additionally, self-reported sociodemographic items were collected at baseline (T1).

#### Participant Matching

Participants from the medical marijuana group were age-, sex-, and race-matched to participants in the control group. Variables used for participant matching between conditions were age, race, and sex. Age match was achieved by starting with the age of the medical marijuana participant and then identifying a race and sex match that was ±5 years of the participants’ age. As an example, a 62-year-old white male enrolled in the medical marijuana group could be matched with any white male participant in the control group between the ages of 57 and 67. Race was operationalized through self-report from a list of standard US census categories (White, Black or African American, American Indian or Alaska Native, Asian, Native Hawaiian or Other Pacific Islander, or Others) [30]. Sex was operationalized through self-report from a list of 2 categories (male or female). Other self-reported sociodemographic variables such as income, employment status, and education level were not used for participant matching.

#### Primary Outcome

Driving simulator data were collected through the kinematic functionality of the Real Time Technologies Inc. (Royal Oak, MI) immersive and high-fidelity driving simulator (eg, speed in miles per hour and lateral lane positioning). Additionally, a trained evaluator observed the driver’s performance for yes or no error responses using the playback function of the simulator to scrutinize and validate the observed errors.

The drive began with a 5-minute acclimation scenario designed to train drivers on aspects of simulator operation and to help them adjust to driving in a simulator [30]. The simulator acclimation drive addresses lane keeping on straight and curved roads, changing lanes, using side- and rear-view mirrors, and stopping.

After the first 5-minute acclimation scenario, the driver drove the main drive for another 15 minutes. The first 7 minutes of driving included daytime driving, starting in a rural area with ambient traffic, rolling hills, and curves on the road and included occasional lead vehicles, oncoming traffic, and vehicles behind the driver. Part of the drive consisted of straight residential roadways connecting 8 left turns and 2 right turns with minimal ambient traffic. The last 8 minutes of the drive took place in a residential area at low speeds (15‐30 mph) and then progressed to a city at a speed of 30 to 45 mph. This part of the drive occurred in a suburban environment (such as Gainesville, FL) during daytime driving with minimal lead, following, and oncoming traffic, as well as some pedestrians and cyclists. The streets were surrounded by buildings, strip malls, and stores. Two scripted events were randomly presented to the drivers to control learning effects.

- Event 1: to assess the response time during driving, a vehicle is pulled out in front of the driver, and the driver’s behavior is measured by braking reaction time in milliseconds (ms) and swerving (SD of lateral deviation from the center lane). The outcome variable for this event was average gross response time in milliseconds (ms).
- Event 2: next, using an established protocol [31], DA (being aware of the traffic and surroundings while focusing on the critical stimulus) was assessed by a triangle appearing on the screen, and the participants responded by pushing a red button on the vehicle’s dashboard as soon as they observed it (measured in milliseconds and number of omissions and commissions). Four trials were conducted during the event. The outcomes for this event were the average DA reaction time in milliseconds for all 4 trials and the number (range: 0‐4) of DA tasks correctly completed for all 4 trials.

**Figure 1.**
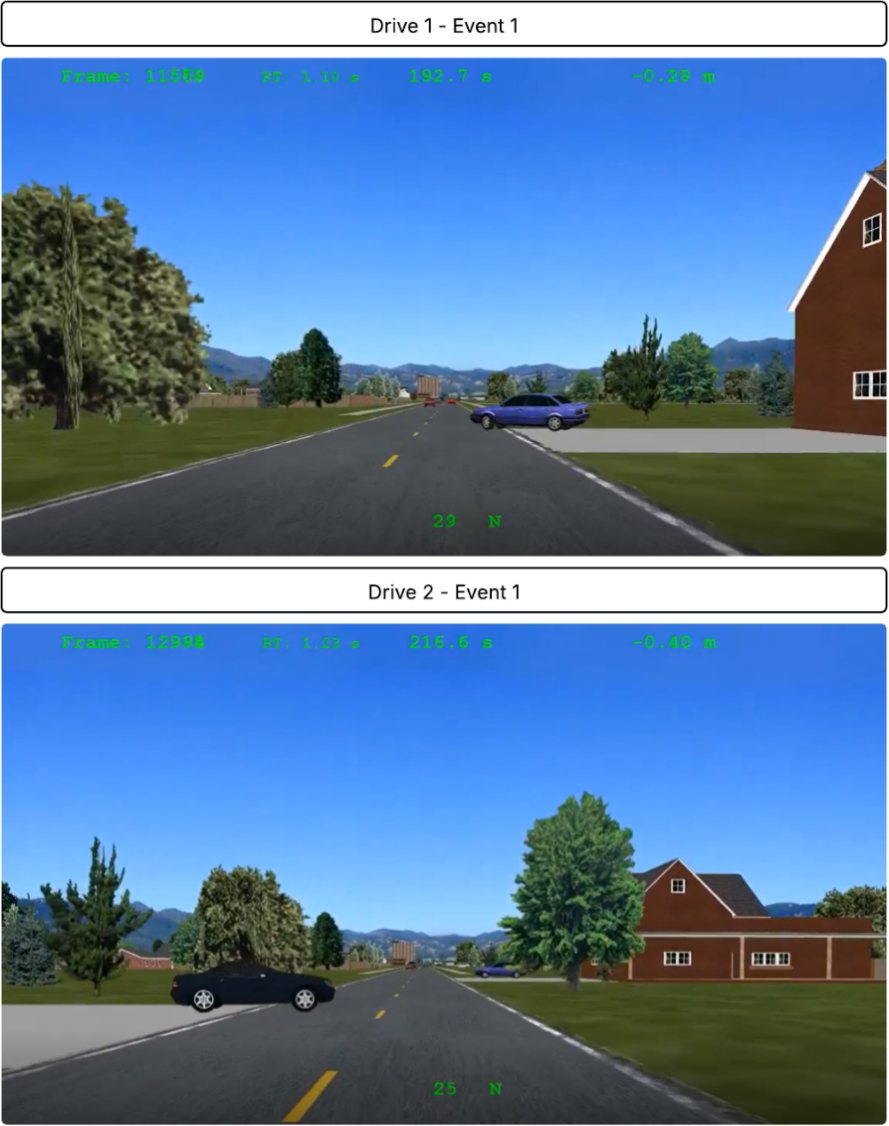
Event 1 differences for drive 1 and drive 2.

**Figure 2.**
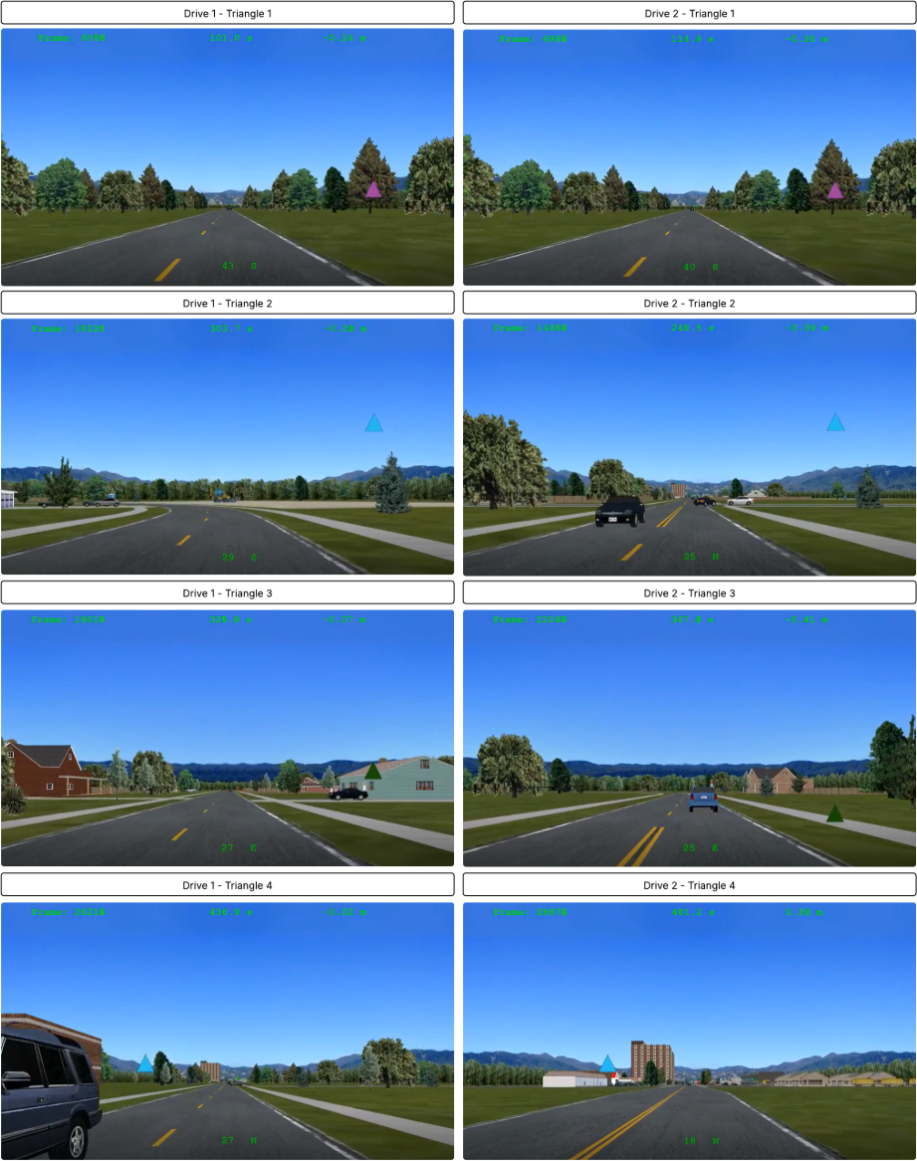
Triangle differences for drive 1 and drive 2.

Participants were randomized to determine which drive (Drive 1 or Drive 2) they would complete at each timepoint (T1 and T2). Events 1 and 2 were identical across both drives except for the following differences. Regarding Event 1, a vehicle pulled out from a driveway on the right for Drive 1 and pulled out from a driveway on the left at Drive 2. See Figure 1 for additional details. Regarding Event 2, the triangle appearances (color, position, and route location) differed throughout each drive; they were matched as closely as possible between the 2 drives but were not strictly identical. See Figure 2 for additional details.

### Analytical Plan

For aim 1, which evaluated the feasibility of recruiting and testing participants, we summarized participation rates at each stage of recruitment and documented reasons for nonparticipation. Issues encountered during driving simulator testing, including simulator sickness and other technical or participant-related factors, were also summarized descriptively.

For aim 2, which explored preliminary associations between medical marijuana initiation and simulated driving performance, we first reported descriptive statistics for potential confounding variables and the focal driving performance outcomes. Baseline differences between the medical marijuana and control groups were examined using independent samples *t* tests for continuous variables and chi-square tests for categorical variables.

For the focal driving performance outcomes, we first examined distributional properties within each group versus time condition. Skewness and kurtosis values were inspected to assess departures from normality. Consistent with recommended guidelines (±1.96) [32], most variables demonstrated acceptable normality, with skewness and kurtosis values within this range except for 1 moderate kurtosis value (2.66).

To examine whether medical marijuana initiation was associated with simulated driving performance, we estimated linear mixed effects models [33,34]. These models included fixed effects for group (medical marijuana vs control), time (baseline vs follow-up), sex, and the group × time interaction, with a random intercept for participant to account for repeated observations within individuals. The group × time interaction was used to test whether changes in driving performance differed between groups. Mixed effects models were selected because they can accommodate unequal sample sizes and missing observations across timepoints and are relatively robust to moderate deviations from normality and unequal variances.

Because this study was designed as a feasibility study with a small sample size, the analyses were considered exploratory. Statistical significance was set at *P*<.05. Effect sizes and 95% CI were reported to aid interpretation of the findings.

## Results

### Participants

**Figure 3.**
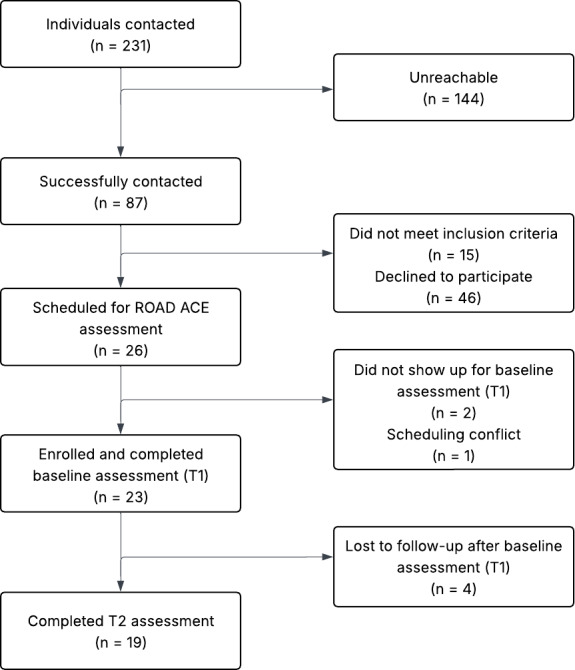
Recruitment and retention flow for the medical marijuana group. ROAD ACE: Reactions of Older Adults While Driving After Cannabis Exposure; T1: timepoint 1; T2: timepoint 2.

**Figure 4.**
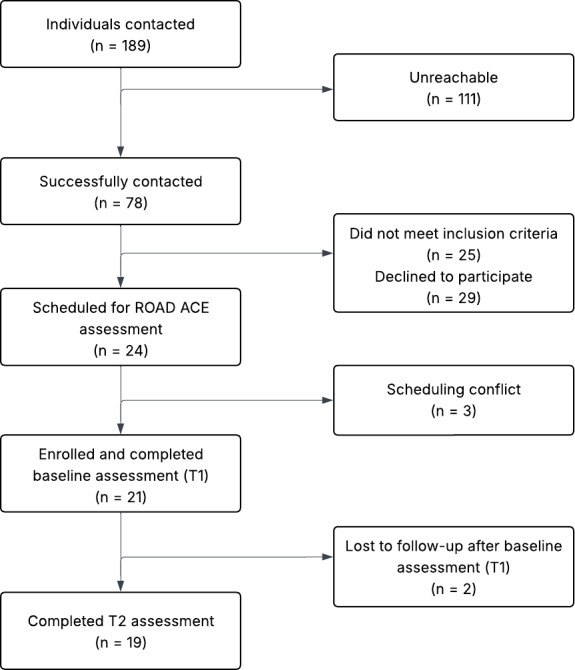
Recruitment and retention flow for the non–marijuana-using control cohort. ROAD ACE: Reactions of Older Adults While Driving After Cannabis Exposure; T1: timepoint 1; T2: timepoint 2.

A total of 231 individuals were contacted for the medical marijuana cohort (Figure 3) and 189 individuals were contacted for the control cohort (Figure 4). Among them, 87 out of 231 (38%) and 78 out of 189 (41%) participants were successfully contacted, respectively. After screening, 61 medical marijuana candidates (15 not meeting inclusion criteria and 46 declining participation) and 54 control candidates (25 not meeting inclusion criteria and 29 declining participation) were excluded. Subsequently, 26 medical marijuana participants and 24 control participants were scheduled for ROAD ACE (Reactions of Older Adults While Driving After Cannabis Exposure) assessment. Of these, 3 medical marijuana candidates (2 no shows and 1 scheduling conflict) and 3 control candidates (scheduling conflicts) did not complete the baseline visit. Ultimately, 23 medical marijuana participants and 21 control participants enrolled and completed the baseline assessment (T1). Following T1, 4 participants in the medical marijuana cohort and 2 participants in the control cohort were lost to follow-up. As a result, 19 participants in each cohort completed the follow-up assessment (T2).

Table 1 presents the descriptive statistics for self-reported sociodemographic variables assessed at baseline (T1) for the entire sample, the medical marijuana group, and the control group, along with test statistics comparing these variables between the two groups. Across the full sample of 44 participants, the mean age was 62.80 (SD 6.62) years, 25 (57%) were male, 41 (93%) were White, and 43 (98%) were non-Hispanic. The median education level was college (4-y degree or university), and the median annual family income was US $30,001 to $60,000. No significant differences were observed between the medical marijuana and the control groups on any sociodemographic variables, confirming that the groups were successfully matched as planned.

**Table 1.** Sociodemographic and psychosocial timepoint 1 (T1) and mental (T1 and timepoint 2 [T2]) and pain (T1 and T2) variables.

Variable	Entire sample (N=44)	Medical marijuana group (n=23)	Control group (n=21)	*t* test or *χ*^2^ test (*df*)	*P* value
Age, mean (SD)	62.8 (6.62)	61.48 (6.42)	64.24 (6.68)	−1.4 (41.28)^a^	.17
Number of individuals in the household, mean (SD)	2.16 (0.99)	2.35 (0.98)	1.95 (0.97)	1.34 (41.7)^a^	.19
Mental status (MMSE-2: BV^b^) T1, mean (SD)	15.3 (0.93)	15.17 (1.15)	15.43 (0.6)	−0.93 (33.63)^a^	.36
Mental status (MMSE-2: BV) T2, mean (SD)	15.1 (0.94)	15.1 (0.91)	15.11 (0.99)	−0.02 (36.3)^a^	.99
Depression (CES-D^c^) T1, mean (SD)	12.52 (9.31)	16.3 (10.23)	8.38 (6.06)	3.16 (36.28)^a^	.99
Depression (CES-D) T2, mean (SD)	11.08 (9.44)	11.75 (9.67)	10.37 (9.41)	0.45 (36.98)^a^	.65
Average pain in past 24 h T1, mean (SD)	3.57(2.27)	5.12 (1.46)	2.73 (2.22)	3.1 (19.84)^a^	<.001
Average pain in past 24 h T2, mean (SD)	3.46 (2.2)	4 (2.33)	3.22 (2.16)	−0.8 (12.6)^a^	.44
Sex, n (%^d^)				1.39^e^ (1)	.24
Male	25 (57)	15 (65)	10 (48)		
Female	19 (43)	8 (35)	11 (52)		
Race, n (%)				0.27^e^ (1)^f^	.61
White	41 (93)	21 (91)	20 (95)		
Black	3 (7)	2 (9)	1 (5)		
Hispanic ethnicity, n (%)				0.93^e^ (1)^f^	.33
No	43 (98)	22 (96)	21 (100)		
Yes	1 (2)	1 (4)	0 (0)		
Education, n (%)				9.56^e^ (6)^f^	.14
Middle school (junior high school) or less	1 (2)	1 (4)	0 (0)		
High school graduate or GED or equivalent	5 (11)	3 (13)	2 (10)		
Junior (2 y) college	5 (11)	3 (13)	2 (10)		
Technical or trade or vocational school	4 (9)	2 (9)	2 (10)		
Some college (4 y degree or university)	11 (25)	9 (39)	2 (10)		
College graduate (4 y degree or university)	9 (20)	2 (9)	7 (33)		
Post college or postgraduate	9 (20)	3 (13)	6 (29)		
Home, n (%)				2.48^e^ (2)^f^	.29
Own or rental home or rental apartment	39 (89)	21 (91)	18 (86)		
Staying at home of family member(s)	3 (7)	2 (9)	1 (5)		
Other	2 (5)	0 (0)	2 (10)		
Employment, n (%)				0.24^e^ (4)^f^	.99
Working full-time	8 (18)	4 (17)	4 (19)		
Working part-time	2 (5)	1 (4)	1 (5)		
Looking for work or unemployed	2 (5)	1 (4)	1 (5)		
Retired	20 (46)	10 (44)	10 (48)		
Disabled, permanently, or temporarily	12 (27)	7 (30)	5 (24)		
Annual family income (US $), n (%)				1.39^e^ (3)	.71
<30,000	12 (27)	5 (22)	7 (33)		
30,001-60,000	14 (32)	9 (39)	5 (24)		
60,001-90,000	6 (14)	3 (13)	3 (14)		
≥90,001	12 (27)	6 (26)	6 (29)		

a *t *test values.

b MMSE-2: BV: Mini-Mental State Examination Second Edition, Brief Version.

c CES-D: the Center for Epidemiologic Studies Depression Scale.

d %: valid percent of participant.

e Chi-square values.

f Results need to be interpreted with caution as less than 80% of cells have expected count less than 5 or minimum expected count less than 1.

### Driving Simulator Testing Issues

Unfortunately, fewer than half of the enrolled participants (18/44, 41%; 12 in the medical marijuana group and 6 in the control group) completed the driving simulator tests at both assessment timepoints. Table 2 presents the detailed reasons for missing data for each group at each assessment time. The primary reason for missing data was simulator sickness, accounting for 22 of 26 cases of missing data at T1 and 19 of 26 at T2. Simulator sickness occurred in 34.8% (8/23) of participants in the marijuana group and 66.7% (14/21) of participants in the control group. This difference did not reach statistical significance (Fisher exact test, odds ratio=3.63, 95% CI 0.92‐15.77; *P*=.07). Because simulator sickness can range from mild to severe, participants were allowed to decide whether to continue the simulated driving task if symptoms occurred. As a result, 8 participants who experienced mild simulator sickness at T1 still attempted the driving task at T2.

**Table 2.** Driving simulator testing issues at timepoint 1 and timepoint 2 for each group.

Reasons for missing data	Timepoint 1 (T1)		Timepoint 2 (T2)	
	Medical marijuana group (n=23), n (%)	Control group (n=21), n (%)	Medical marijuana group (n=23), n (%)	Control group (n=21), n (%)
Drive not completed due to simulator sickness	8 (35)	14 (67)	2 (9)	3 (14)
Drive not attempted due to simulator sickness at T1	0 (0)	0 (0)	5 (22)	9 (43)
Participant requested to stop	1 (4)	0 (0)	1 (4)	0 (0)
Participant size could not be accommodated	0 (0)	1 (5)	0 (0)	1 (5)
Technical problem with driving simulator	2 (9)	0 (0)	0 (0)	0 (0)
Lost to follow-up	0 (0)	0 (0)	3 (13)	0 (0)
Withdrew from study	0 (0)	0 (0)	0 (0)	2 (10)
Completed drives at T1 and T2 (no missing data)	12 (52)	6 (29)	12 (52)	6 (29)

Consequently, some participants contributed partial driving data. Among the 26 participants with missing data, 12 (46%) had partial data at T1 and 5 (19%) had partial data at T2. The valid sample size for the 6 focal DA outcomes (ie, brake response time, reaction time in 4 DA scenarios, and number of responses to DA events) ranged from 12 to 18 across times among the marijuana group and from 7 to 12 across times among the control group (Table 3).

**Table 3.** Descriptives for driving test variables at timepoint 1 and timepoint 2.

Driving test variables	Medical marijuana group						Control group					
	N^a^	Male	Female	Mean (SD)	Skewness	Kurtosis	N	Male	Female	Mean (SD)	Skewness	Kurtosis
Timepoint 1 (T1)												
Trigger_RT^b^_T1	18	13	5	1.22 (0.28)	0.18	−1.56	12	7	5	1.24 (0.21)	0.31	−1.46
DA^c^1_RT_T1	18	13	5	1.77 (0.57)	1.32	2.66	12	7	5	1.95 (0.53)	0.39	−1.39
DA2_RT_T1	16	12	4	6.05 (3.5)	0.09	−1.88	11	7	4	8.55 (2.69)	−1.18	−0.34
DA3_RT_T1	14	10	4	4.07 (3.71)	0.79	−1.36	10	7	3	4.92 (3.79)	0.46	−1.78
DA4_RT_T1	13	10	3	7.22 (3.27)	−0.55	−1.53	9	7	2	5.69 (3.56)	0.23	−1.88
DA_Omission_T1	13	10	3	0.85 (0.69)	0.16	−1.09	10	8	2	1.4 (1.07)	0.23	−1.42
Timepoint (T2)												
Trigger_RT_T2	15	11	4	1.24 (0.23)	0.44	−0.78	7	7	0	1.29 (0.4)	0.38	−1.68
DA1_RT_T2	15	11	4	1.87 (0.34)	0.06	−1.3	7	6	1	1.67 (0.26)	0.22	−1.31
DA2_RT_T2	14	10	4	5.38 (3.28)	0.48	−1.52	7	7	0	8.12 (3.26)	−0.76	−1.58
DA3_RT_T2	14	10	4	2.57 (1.63)	1.55	1.16	7	7	0	5.79 (4.32)	−0.05	−2.14
DA4_RT_T2	12	9	3	5.38 (3.52)	0.21	−1.8	7	7	0	5.36 (3.24)	0.07	−1.83
DA_Omission_T2	12	9	3	0.58 (0.67)	0.56	−0.97	7	7	0	1.43 (0.98)	0.17	−1.33

a N: number of participants.

b RT: reaction time.

c DA: divided attention.

Little test indicated that missing data for these focal variables were missing completely at random (*χ*²_67_=64.23, *P*=.57). Attrition analyses comparing participants with complete data at T2 (n=18) and those with missing data (n=26) showed no significant differences in sociodemographic or mental health variables at T1, except that participants with missing data were more likely to be female (*χ*²_1_=8.73, *P*<.01). Thus, in analyses for Aim 2, sex was included as a covariate.

### Confounding Variables

#### Mental and Pain Variables

The medical marijuana and control groups were not significantly different in any mental and pain variables, except that (1) the marijuana group (mean 16.30, SD 10.23) had higher levels of depression than the control group (mean 8.38, SD 6.06; *t*_36.28_=3.16, *P*<.01) at T1; however, such group differences became nonsignificant at T2 (*t*_36.98_=3.16, *P*=.65); (2) marijuana group (mean 5.12, SD 1.46) had higher levels of average pain in the past 24 hours than the control group (mean 2.73, SD 2.22; *t*_19.84_=3.1, *P*<.01) at T1; however, such group differences became significant at T2, (*t*_12.6_=0.8, *P*=.44).

#### Driving Habits Assessed at T1

All participants drove in their daily lives, with an average of self-reported 5.14 (SD 2.02) days per week and self-reported 105.42 (SD 101.31) miles per week. The medical marijuana and control groups were similar in average driving days per week (*t*_42_=−1.07, *P*=.29) and miles per week (*t*_41_=1.08, *P*=.29). No one suggested limiting or stopping driving during the past year. Most participants always wore a seat belt (n=42, 96%) and had a very low or low risk of crashes based on the Useful Field of View (n=41, 93%). During the past 3 months, all participants drove when it was raining or alone; most participants drove on interstates or expressways (n=42, 96%), on high-traffic roads (n=43, 98%), in rush-hour traffic (n=42, 96%), or at night (n=42, 96%). During the past year, all participants had driven to neighborhood towns, most participants had driven to more distant towns (n=40, 91%), and slightly over half of the participants were driven outside Florida (n=25, 57%).

#### Classification of Marijuana Use Patterns

Qualitative interviews were conducted at timepoint 2 (T2) to obtain data on 7-day patterns of medical marijuana use. Use categories were based on the number of products used, routes of administration (eg, edibles, smokable flowers, oral, sublingual, and concentrates), quantity of use, and frequency of use. The participants were classified as no use, light use, moderate use, or heavy use by 2 independent coders. All conflicts were resolved using a third coder through a process of consensus. Forty-two percent of the medical marijuana group was classified as heavy use, 16% as moderate use, 32% as light use, and 10% reported no use past 7 days. All participants in the control group reported no use past 7 days.

#### Urine Test of Drugs at T2

Urine test results showed that 17 out of 19 (89%) participants were positive for marijuana in the medical marijuana group compared to no participants in the control group. Only a few patients received other drugs, and the pattern of other drugs was consistent between the medical marijuana and control groups. Further details are presented in Table 4.

**Table 4.** Urine test results of the presence of drugs at timepoint 2 (N=19).

Drug	Medical marijuana group, n (%)^a^^,^^b^	Control group, n (%)^a^^,^^b^
Marijuana	17 (89)	0 (0)
Buprenorphine	3 (16)	1 (5)
Methadone metabolites	0 (0)	0 (0)
Tricyclic antidepressants	4 (21)	3 (16)
Barbiturates	0 (0)	0 (0)
Benzodiazepines	2 (11)	2 (11)
Methadone	0 (0)	0 (0)
Amphetamine	1 (5)	0 (0)
Morphine	1 (5)	1 (5)
Oxycodone	2 (11)	1 (5)
Ecstasy	0 (0)	0 (0)
Cocaine	0 (0)	0 (0)
Phencyclidine (PCP)	0 (0)	0 (0)
Methamphetamine	0 (0)	0 (0)

a n: number of participants with positive screening for the drug.

b %: valid percentage of the sample.

### Driving Performance

Descriptive statistics of the 6 driving test variables are presented in Table 3. Linear mixed effects models were used to examine whether changes in driving performance differed between the medical marijuana and control groups over time, controlling for sex. The group×time interaction was not statistically significant for any driving performance outcome (Table 5). Effect sizes for the interaction terms were small to moderate (|*d*|=0.02‐0.66), and all CIs included zero, suggesting no clear evidence that initiation of medical marijuana was associated with changes in simulated driving performance relative to controls over the study period.

**Table 5.** Group×time interaction effects on simulated driving performance outcomes.^a^

Outcome	*β* (estimate) (95% CI)	*P* value	Cohen *d*
Trigger_RT^b^	−0.01 (−0.34 to 0.32)	.97	−0.02
DA1_RT^c^	0.30 (−0.23 to 0.83)	.25	0.54
DA2_RT	−0.35 (−4.48 to 3.78)	.86	−0.11
DA3_RT	−2.47 (−6.76 to 1.82)	.25	−0.66
DA4_RT	−1.90 (−6.37 to 2.58)	.40	−0.56
DA_Omission	−0.38 (−1.49 to 0.72)	.48	−0.44

a Estimates represent the group×time interaction from linear mixed effects models controlling for sex. Negative values indicate larger decreases in reaction time or omission errors for the medical marijuana group relative to controls from baseline to follow-up. CI that include zero indicate nonsignificant effects.

b RT: reaction time.

c DA: divided attention.

## Discussion

### Principal Findings

This study assessed the feasibility of recruiting patients using medical marijuana prior to the initiation of medical marijuana treatment and age-, race-, and sex-matched controls in a driving simulation study to assess the effects of medical marijuana use on real-world outcomes using driving performance. This feasibility study examined participants under real-world conditions to develop a knowledge base needed for guidelines and best practices that currently do not exist for medical marijuana use in this population. The 7-day marijuana use reflected a spectrum from no use to heavy use, with most participants reporting moderate to heavy use over the past 7 days.

Recruitment efforts produced mixed results; the study successfully recruited the majority of the initial a priori sample size proposed but did not fully meet the goal of age-, race-, and sex-matched controls. Age match was achieved by expanding the criteria to ±5 years of medical marijuana group participant age in matching to control participants, as opposed to ±1 year as initially proposed. We used a multiprong approach to recruit participants in both the medical marijuana and control study conditions. Obstacles that reduced the feasibility of this study included the narrow inclusion criteria for control participants (age, race, and sex matching) and COVID-19 restrictions, which limited the access to potential populations of adults aged 50 years and older, who would qualify as control participants. Although these factors reduced the feasibility of recruiting participants, recruitment efforts were successful for the medical marijuana group participants, particularly because of strong stakeholder engagement and our recruitment partnership with the MMTC.

Current research indicates that marijuana users experience cognitive impairment in areas such as psychomotor speed and DA [35-40]. Our work suggests a more nuanced story; however, all interpretations from this data must be done with great caution due to the limited sample size. Specifically, regarding marijuana initiation, we did not find any evidence that initiation of medical marijuana was associated with changes in simulated driving performance relative to controls over the study period. The data also showed that depressive symptoms significantly decreased in the medical marijuana group. Further work is needed to understand whether medical marijuana use creates a long-term impact on psychomotor functioning that affects real-world tasks, such as response time, attention, and executive functions, all of which are critical for driving.

As expected [41], simulator sickness occurred in numerous cases, leading to the majority of missing data in the study. Data show that older females are more susceptible to simulator sickness than their male counterparts [41]—as such, the mitigation of simulator sickness remains a high priority for any future work with driving simulators. Additional emphasis must be placed on ensuring that the participants understand the importance of implementing the mitigation strategies, given that experiencing simulator sickness symptoms was the primary reason the participants did not complete the driving scenarios. Our findings suggest that when using an immersive, full-body cab driving simulator, it is imperative that it provide both sympathetic and parasympathetic feedback. The limitation of our simulator is that it did not provide kinetic feedback for participants, which led to greater instances of simulator sickness. The substantial proportion of missing data attributable to simulator sickness suggests that simulator-based assessments may present challenges for this population and that more realistic on-road driving assessments in line with the national driving test standard may be a more suitable approach in future studies. An open-road driving task is available and enables a more accurate assessment of older drivers in a heterogeneous group [42].

The present feasibility study demonstrated the ability to prospectively recruit the target population, successfully implement the study protocol, and retain participants in this feasibility study. First, many epidemiological studies on marijuana and driving rely primarily on self-reported marijuana use and driving behaviors [35,43]. Our study was able to prospectively track marijuana use by using multiple data sources and a simulated driving task. Second, we focused on medical marijuana use rather than on recreational marijuana use, which is more prevalent among older adults. Driving studies generally focus on recreational marijuana use [44], which is not comparable to medical marijuana due to the lack of regulatory oversight and creates an additional challenge to measuring and tracking use. Third, our study adds data to the literature above and beyond just the presence or absence of marijuana, as medical marijuana group participants drove within 1 month of starting medical marijuana, and use patterns were clearly classified. Studies that report toxicology at crash sites can only report the presence or absence of tetrahydrocannabinol in the system, but they cannot tell us how proximal marijuana use was to the crash, and if the use pattern, a modifiable risk factor, had any effect. The limitations of the study include difficulty recruiting control participants, which has been particularly challenging during the COVID-19 pandemic. Simulator sickness remains an issue to be managed, as this represented the primary driver of missing data, which may introduce potential bias in this small feasibility study. Therefore, the findings regarding the relationship between medical marijuana initiation and changes in driving performance should be interpreted cautiously and considered preliminary at present. The absence of clinical variables such as sleep quality, psychoactive medication use, and comorbidity burden limits the ability to examine their potential role as unmeasured confounders. Finally, while the study is representative of medical marijuana patients in Florida, the generalizability of the results is limited due to the low enrollment of racial or ethnic minority participants. Although Florida’s Office of Medical Marijuana Use does not publish sociodemographic data on its patient registry, current literature suggests that white patients are overrepresented while patients of racial and ethnic minorities are underrepresented in Florida [45,46] and is consistent with medical marijuana research on a national level [45,46]. Potential barriers to diverse recruitment include financial burden not covered by any insurance associated with acquiring and maintaining a medical marijuana card, historic stigma, and disparities in access to and education about medical marijuana care.

### Conclusions

This study is the first to prospectively recruit mid-to-late-life medical marijuana users and examine their driving performance in an immersive full-body cab driving simulator under real-world conditions. The next step in this line of research is a study that systematically assesses driving performance in a rigorous and ecologically valid manner, accounting for long-term medical marijuana use in adults 50 years and older who endorse chronic nonmalignant pain. Fitness-to-drive must be measured using an open-road course under the observation of a Certified Driver Rehabilitation Specialist trained to evaluate impaired driving in a test vehicle with the required evaluator controls (ie, eye-check mirrors, auxiliary brake, and auxiliary gas) to maneuver the car and prevent any adverse events, including near misses or crashes, if any dangerous situations arise. As medical marijuana use increases, data are needed to develop risk screening, best practice guidelines, and relevant interventions for this population.

## Supplementary material

10.2196/79735 Checklist 1 STROBE checklist (cohort study)—manuscript mapping.

## Abbreviations

DA: divided attention
IRB: institutional review board
MMSE-2: BV: Mini-Mental State Examination Second Edition, Brief Version
MMTC: Medical Marijuana Treatment Clinics
ROAD ACE: Reactions of Older Adults while Driving After Cannabis Exposure
STROBE: Strengthening the Reporting of Observational Studies in Epidemiology
T1: timepoint 1
T2: timepoint 2
