# Supplementary material for: Medical Marijuana Initiation and Simulated Driving Performance Among Mid-to-Late-Life Adults With Chronic Pain: Prospective Observational Feasibility Cohort Study With Matched Controls
Source: JMIR Form Res. 2026 May 8;10:e79735. doi: 10.2196/79735 (PMC13155503; doi:10.2196/79735)
Supplement: Checklist 1 [file formative-v10-e79735-s001.docx]

# STROBE Checklist (Cohort Study) – Manuscript Mapping

| **Section** | **Item** | **Recommendation** | **Section (Page Number)** |
| --- | --- | --- | --- |
| Title/Abstract | 1a | Indicate the study’s design with a commonly used term in the title/abstract | Title Page (1) |
| Title/Abstract | 1b | Informative and balanced summary | Abstract (2) |
| Introduction | 2 | Background/rationale | Introduction (3) |
| Introduction | 3 | Objectives/hypotheses | Abstract (2) & Introduction (3) |
| Methods | 4 | Key elements of study design | Methods (4) |
| Methods | 5 | Setting, locations, dates | Methods (4), Procedure, (7), & Timeline (7-8) |
| Methods | 6a | Eligibility criteria and selection | Methods (4) |
| Methods | 6b | Matching criteria and numbers | Methods (4), Recruitment (7-8), & Table 1 (14) |
| Methods | 7 | Define outcomes/exposures/confounders | Methods (4), Measures (8), Primary Outcome (9) & Analytical Plan (12) |
| Methods | 8 | Data sources/measurement | Methods (4), Measures (8), & Primary Outcome (9) |
| Methods | 9 | Address bias | Methods (4), Discussion (20) |
| Methods | 10 | Study size | Methods (4-6), Analytical Plan (12) |
| Methods | 11 | Quantitative variables | Methods (8), Analytical Plan (12) |
| Methods | 12a | Statistical methods | Methods, Analytical Plan (12) |
| Methods | 12c | Missing data handling | Analytical Plan (12), Results (13) |
| Methods | 12e | Sensitivity analyses | N/A due to lack of model significance |
| Results | 13a | Numbers at each stage | Results (12-13), Figure 1 (5), & Figure 2 (6) |
| Results | 13b | Reasons for non-participation | Results (12-13), Table 2 (15) |
| Results | 14a | Participant characteristics | Results (12-13), Table 1 (14-15) |
| Results | 14b | Missing data per variable | Results (13), Table 1 (14-15) |
| Results | 15 | Outcome data | Results, Table 3 (16) |
| Results | 16a | Main results (unadjusted/adjusted if applicable) | Results, Table 3 (16) |
| Discussion | 18 | Key results | Discussion (18-20) |
| Discussion | 19 | Limitations | Discussion (19-20) |
| Discussion | 20 | Interpretation | Discussion (19-20) |
| Discussion | 21 | Generalizability | Discussion (20) |
| Other | 22 | Funding | Funding Statement (21) |
